# Stability of conventional vs. Pickering emulsions using ovalbumin-anionic starch complexes for spray-dried lipid microcapsules

**DOI:** 10.1016/j.fochx.2025.103102

**Published:** 2025-10-02

**Authors:** Rabia Ramzan, Zafarullah Muhammad, Fahao Shi, Wenjing Tang, Zhou Wang, Nazia Khalid, Song Li, Ana Chen

**Affiliations:** a School of Biological and Food Engineering, Anhui Polytechnic University, Beijing Middle Road, Wuhu, Anhui 241000, People's Republic of China; b Department of Food Science and Technology, Government College Women University, Faisalabad 38860, Pakistan; c College of Agriculture and Food Engineering, Baise University, Baise, Guangxi Zhuang Autonomous Region 533000, People's Republic of China

**Keywords:** Anionic starch, Ovalbumin, Polyelectrolytic complexes, Pickering emulsions, Microcapsules, Oxidative stability

## Abstract

The utilization of oils rich in polyunsaturated fatty acids can be enhanced by transforming them into spray-dried powdered microcapsules to inhibit oxidation. The functional characteristics of biopolymers, such as proteins and modified starch, may be combined to generate superior protective matrices in the form of stable emulsions. In this study, polyelectrolyte complexes of anionic starch (AS) and ovalbumin (OVA) at varying OVA/AS ratios (1:0, 0:1, 1:0.5, 1:1, 2:1, 3:1) were used to spray-dry and microencapsulate chia seed oil. AS and OVA interaction created viable polyelectrolyte complexes when turbidity was measured at varying OVA/AS ratios across pH 1–10. Higher AS to OVA ratios improved antioxidant properties, wettability (74.9°-84.1°), hydrophobicity (288.67 ± 6.06 μg/g-1980 ± 8.78 μg/g), and emulsification. Moreover, the microcapsules showed the highest encapsulation efficiency (83.94 ± 1.05 %), total oil contents (68.90 ± 0.50 %), and the lowest surface oil (11.06 ± 1.37 % at 1:1 OVA) using complexes for Pickering emulsions through homogenization and spray drying. These microcapsules demonstrated reduced peroxide values after 30 d of storage at 50 °C (56.27 ± 0.68 meqO_2_/kg), 80 % humidity (84.37 ± 0.68 meqO_2_/kg), or light (54.84 ± 0.71meqO_2_/kg) compared to OVA-stabilized emulsion microcapsules. OVA/AS polyelectrolyte complex-stabilized Pickering emulsions may facilitate the production of stable oil microcapsules for food applications.

## Highlights

• Fabricating the antioxidant polyelectrolyte complex with ovalbumin and anionic starch.
• Formulating Pickering emulsions of chia seed oil stabilized by varying OVA:AS ratios.
• Developing the spray-dried lipid microcapsules containing chia seed oil.
• Enhancing the oxidation stability of microcapsules under different T and RH conditions.

## 1 Introduction

Health is the most crucial aspect of human life, and food is essential for sustaining health. The food industry and scientific community are escalating their research interest in exploring the nutritional and nutraceutical properties and benefits of plant oil-based functional ingredients to formulate functional foods for the vegan or vegetarian population worldwide (Castejón et al., 2021). Oils with high amounts of Omega-3 Polyunsaturated fatty acids (PUFAs) are the functional ingredients used in food products. Because PUFAs have health-promoting effects regarding anti-inflammation, cognitive health, anticancer, and non-communicable diseases (NCDs) such as cardiovascular disorders, maintaining blood pressure through retaining the levels of triglycerides and vasodilation, and healthy development of the visual systems and brain in infants (Borsoi et al., 2023). Among the essential fatty acids, omega-3 α-linolenic acid (ALA) is considered a parent fatty acid from which essential omega-3 PUFAs are derived (Alvarez et al., 2024). Fish is the primary source of ALA, but due to the toxicity of heavy metals (mercury and copper), synthetic organic contaminants such as PCBs (polychlorinated biphenyls) in some fish species, and the increasing global market of vegan foods, it is essential to introduce sustainable and alternative sources of omega-3 fatty acids (Castejón et al., 2021). Compared to fish oil, Chia seed oil is a plant-based source that is a renewable, cost-effective, and sustainable vegetable source of omega-3 PUFAs due to the rich content of α-linolenic acid (ALA). It is doubtless that chia seed oil has excellent nutraceutical and nutritional value due to its high degree of PUFAs, but it also increases its vulnerability to lipid oxidation (Rosenberg et al., 2018). The oxidation products impart unpleasant flavors and odors due to the formation of alcohols and volatile ketones in foods. Consequently, due to the lower oxidative stability and hydrophobicity, adding or fortifying omega-3 oils in hydrophilic functional foods is a critical challenge for the food industry.

Spray-dried lipid microcapsules are used extensively in the food sector. Due to their high oxidation susceptibility, their microencapsulation is still difficult. To resolve this problem, a prevalent technique involves reducing the surface oil content of the microcapsules by improving process parameters or incorporating additional natural or synthetic antioxidants into either the aqueous or oil phase (Espinosa-Andrews et al., 2023). According to (Daza et al., 2025), microencapsulation of bioactive lipids, which usually involves emulsification and spray drying procedures, may make handling and storage easier, increase water-dispersibility, and prevent oxidation. This approach, however, incurs additional costs and may adversely affect the quality of end products by influencing the drying process of emulsions. Pickering emulsions provide a viable approach to enhancing the oxidative stability of PUFA microcapsules through alterations in the interfacial layer. They utilize solid particles for stabilization in place of amphipathic (conventional) surfactants and are distinguished by their diverse functionalities and types of stabilizers (Gu et al., 2024).

The oil-water interface in food emulsions is critical for initiating lipid oxidation, and the placement of antioxidants at this interface may effectively mitigate lipid oxidation (Copado et al., 2021; Gu et al., 2024). As a result, stabilized Pickering emulsions with antioxidant properties are projected to be effective options for the safe, efficient, and economical development of the PUFA microcapsules; these microcapsules exhibit resistance to oxidative stress. Egg protein acts as a natural emulsifier, crucial in creating and maintaining high-internal-phase emulsions, such as those found in mayonnaise and salad dressings. Ovalbumin makes up about 65 % of the protein in egg whites and is extensively used in food technology due to its stabilizing and emulsifying characteristics. Furthermore, ovalbumin has been shown to possess immunomodulatory, anticarcinogenic, antimutagenic, and antioxidant (protection of docosahexaenoic acid and linoleic acid) potentials. Despite having superior interfacial and emulsifying qualities, OVA is unable to provide the emulsions with long-term physical stability during further processing and storage (Liu et al., 2025; D. Wang et al., 2024).

So, in his context, there is a dire need to introduce natural emulsifiers to be introduced in the food system. Among these, starch could be the most suitable choice due to its natural abundance, renewability, and cost-effectiveness. Chinese water chestnut (*Eleocharis tuberosa*) belongs to the Cyperaceae family and is abundantly found in oriental countries, especially in southern China (Muhammad, Ramzan, Abdullah Abbas, et al., 2025). Furthermore, the modified *Eleocharis tuberosa* starches (ETS) based on octyl succinic anhydride (OSA) have a unique anionic character that may lead to better antioxidant activity. As a result, Pickering emulsions stabilized by nanoparticles with anionic ETS could provide enhanced protection for encapsulated oils and be employed to develop oxidation-resistant microcapsules (Muhammad, Ramzan, Zhang, & Zhang, 2025).

Complexes of polymers and proteins have been utilized to create protective wall materials against lipid oxidation and to encapsulate live cells, flavorings, cosmetic additives, and bioactive chemicals based on lipids (Gu et al., 2024; D. Wang et al., 2024). The fabrication of the Polysaccharide-based nanoparticles could be done in various ways, including hydrophobic modification, self-aggregation, and ionic gelation. Notably, polyelectrolyte complexation with proteins warrants special attention due to the ionic nature of starch and the simplicity of the method (Sharkawy et al., 2020). Several studies have been carried out to produce stabilized Pickering emulsions containing cationic polysaccharide/protein polyelectrolytes. Still, there is no study available in which anionic polysaccharide (AS)/protein (ovalbumin) polyelectrolyte complex stabilized Pickering emulsions containing encapsulated PUFAs from chia seed oil were subjected to spray drying.

In the present study, we have produced stabilized chia seed oil Pickering emulsions using modified *Eleocharis tuberosa* as an anionic starch (AS) and cationic ovalbumin (OVA) polyelectrolyte complexes to produce the oxidatively stable spray-dried microcapsules. Based on the previous discussion, it could be hypothesized that AS/OVA complexes could be used to enhance the stability of chia seed oil Pickering emulsions as well as to produce spray-dried PUFA microcapsules with enhanced oxidative stability. To test this hypothesis, physicochemical, structural, and morphological studies of the microcapsules were conducted. Overall, this study aimed to relate the interactions of AS and OVA at different ratios to produce stable conventional and Pickering emulsions containing chia seed oil and integrating AS/OVA polyelectrolytic complexes as stabilizing agents, followed by spray-drying to create microcapsules with enhanced resistance to oxidation. The present study is expected to help develop non-conventional sources-based, oxidative-stable microcapsules containing functional ingredients, which would enable the feasible integration of vegetable oil PUFAs to produce enriched functional food and pharmaceutical products.

## 2 Materials and methods

### 2.1 Materials

A 3 % OSA modified *Eleocharis tuberosa* (Chinese water chestnut) starch was taken from the study by Muhammad, Ramzan, Abdullah Abbas, et al. (2025). A 3 % OSA modified starch contains negatively charged carboxyl groups, due to which the modified starch gained negative charge at a specific pH (∼4.8), and we termed it an anionic starch (AS) with a degree of acetylation of 95 % and viscosity of 100–200 mPa·s. The egg white o*v*albumin protein (OVA) was procured from the Cusabio Technology Co., Ltd. (Wuhan, China). Chia seed oil was purchased from the Shanghai Taisun Pharmaceutical Co., Ltd. (Shanghai, China), and it was characterized for an initial peroxide *v*alue (POV) of 48.75 meq/kg. Throughout the in*v*estigation, deionized water was used consistently, and all other chemicals used were of analytical grade.

### 2.2 Fabrication of ovalbumin (OVA):anionic starch (AS) polyelectrolyte complexes and their characterization

#### 2.2.1 The complexes preparation

A 3.5 % (*w*/*v*) solution was produced by dissolving OVA in deionized water and suspending 3.5 g AS in a 100 mL of 1.5 % (*v*/v) solution of acetic acid, which was then magnetically stirred for 5 h at 500 r/min to generate stabilized and properly solubilized 3.5 % (w/v) solution of AS in acetic acid. To enable the formation of OVA:AS polyelectrolytic complexes, the two solutions were then combined in volume ratios of 1:0, 0:1, 1:05, 1:1, 2:1, or 3:1, brought to pH 5.0 using either NaOH or HCl solution, and allowed to stand for 30 min. After the development of the association, the samples were evaluated for the turbidity test and then freeze-dried.

#### 2.2.2 The turbidity measurements

The prepared suspension was immediately diluted 30 times using distilled water upon the completion of the OVA:AS association. And then absorbance measurements were taken at 600 nm by loading samples into a spectrophotometer (UV-2000, Unico, Shanghai, China). Whereas deionized water was used as a control.

### 2.3 FT-IR spectroscopy

Following the method as described by Lopez-Silva et al. (2019), the functional groups of AS starches with 3 % OSA concentrations and OVA and OVA:AS polyelectrolyte complexes (1:1) were characterized by FTIR spectroscopic analysis using a Bruker Vertex 76 FTIR spectrophotometer (Bruker Optik GmbH, Ettlingen, Germany). The apparatus has a diamond crystal with a 45° angle of incidence, linked to a mercury cadmium telluride detector, a KBr beam splitter, and a Platinum Diamond ATR accessory. To collect the spectra in a wavenumber range of 4000–500 cm^−1^, 32 scans were carried out at a resolution of 4 cm^−1^. An analysis of the spectra was conducted using Thermo Scientific OMNIC FTIR software (Thermo Scientific Corporation, Shanghai, China).

### 2.4 Quantification of the antioxidant acti*v*ities

#### 2.4.1 Measuring the free radical scavenging activity of DPPH

With a slighter modification, a DPPH technique, which was previously described by Tuesta-Chavez et al. (2022), was used to assess the samples' capacity to scavenge free radicals. In short, distilled water was mixed with AS, OVA, and their freeze-dried complexes individually to create solutions that were 0.1 % (*w*/*v*). Afterward, 2.4 mL of 0.1 mmol/L DPPH, 1.1 mL of distilled water, and 0.5 mL of the solution were combined. Using the same method, 0.5 mL of distilled water was added to prepare the control instead of the complex solutions. The absorbance at 517 nm was measured after extensive mixing, and Eq. 1 was used to determine the DPPH free radical scavenging rate:

(1) $$\mathrm{DPPH free radical scavenging rate}\;\left(\%\right)=\frac{A_{0}-A_{1}}{A_{0}}\times 100$$

A_0_ and A_1_ represent the absorbance of the control and the absorbance of the samples, respectively.

#### 2.4.2 Ferric reducing antioxidant power (FRAP)

Following the exact instructions given in the manual, the BC1320 T-AOC test Kit (Solarbio Science and Technology Co., Ltd., Beijing, China) was used to conduct the FRAP test. The results, which were represented as mmol FeSO_4_ equivalents, were determined using the linear calibration curves.

### 2.5 The surface hydrophobicity measurements

8-Anilino-1-naphthalenesulfonic acid (ANS) was used as a hydrophobic probe to test the surface hydrophobicity of OVA:AS polyelectrolyte complexes. This approach was slightly modified from that provided by Qin et al. (2017). The phosphate buffer solution (0.01 mol/L, pH 7.0) was used to continually dilute the OVA:AS polyelectrolyte complexes dispersions in order to prepare the samples (0.125–0.5 mg/mL). Then, 4 mL of complex solutions were combined with 40 μL of 8.0 mmol/L ANS solutions. The fluorescence intensity was measured using a Hitachi F-4600 fluorescence spectrophotometer (Tokyo, Japan). The emission and excitation wavelengths were 470 nm and 390 nm respectively, while the emission and excitation slit widths were fixed at 5 nm.

### 2.6 Contact angle (θ) measurements

In accordance with the described method of Qin et al. (2017), contact angles of OVA:AS polyelectrolyte complexes were determined. The contact angles of the freeze-dried samples were calculated using a Data Physics microanalyzer (OCA 15 EC, Germany). A high-speed video camera was built into the device. To prepare the samples for the contact angle measurement, the samples were cut into slices (2 mm) with the help of a tablet press. After placing the slice in a glass container, a drop of Milli-Q water (3 μL) was dropped over its surface. After that, a high-speed camera was used to capture a picture of the contact surface.

### 2.7 Emulsifying properties measurements

The turbidimetric approach was used to assess the emulsion stability and emulsification activity (Q. Wang et al., 2022). The high-speed homogenizer (PT 1200E, Switzerland) was used to homogenize the 16 mL of sample solutions and 4 mL of chia seed oil at 13000 r/min. After homogenization, a 20 μL aliquot of the emulsions was obtained from the bottom of the container and mixed with 5 mL of sodium dodecyl sulfate solution at the respective intervals of 0 and 20 min. The UV–vis spectrophotometer was used to measure the absorbance of the samples at 500 nm. Eqs. (2), (3) were used to determine the emulsion stability index (ESI) and emulsifying activity index (EAI), respectively.

(2) $$\mathrm{ESI}\;\left(\min\right)=\frac{A_{0}}{A_{0}-A_{20}}\times \mathrm{\Delta t}$$

(3) EAIm2g=2×2.303×A0C×1−∅×104×N

In these equations, A_0_ indicates the emulsion's absorbance after homogenization, N represents the dilution factor, C indicates the OVA-AS complexes' concentration, Ø is the emulsion's volume fraction of chia seed oil (0.25), and A_20_ denotes the volume of the emulsion after 20 min.

### 2.8 Development of conventional and pickering emulsions of chia oil by stabilizing with OVA:AS polyelectrolyte complexes

#### 2.8.1 Preparation of the emulsions

After formulating the OVA:AS polyelectrolyte complexes, Chia seed oil was introduced into the suspensions to establish specific mass ratios of complexes 1:0.5, 1:1, 2:1, and 3:1 for Pickering emulsions. Initially, these mixtures were subjected to dispersion using a Scientz-950 E Basic disperser (Ningbo Scientz Biotechnology Co., Ltd., Ningbo, China) operating at 16000 rpm for a duration of 5 mint to form coarse emulsions. Subsequently, they were processed twice through a JN-020C high-pressure homogenizer (Shanghai Donghua High Pressure Homogenizer Co., Ltd., Shanghai, China) at a pressure of 600 bar to achieve fine emulsions. As a control, conventional emulsions 1:0 and 0:1 stabilized solely by OVA and AS were prepared using the same method, respectively. In 1:0, 1 is the concentration of OVA, whereas in 0:1, 1 is the concentration of AS in the conventional emulsions.

#### 2.8.2 Zeta ζ-potential measurements

The determination of the ζ-potential of Chia seed oil emulsions stabilized by OVA:AS polyelectrolyte complexes was carried out by following the method. The suspension was placed into a Zetasizer Nano Series device after 100 times diluting the samples using distilled water (Zetasizer Nano-ZS90, Malvern Instruments, Worcestershire, UK). The zeta potential was assessed by placing the materials into capillary cells. Before taking measurements, the samples were inserted into the test cell and diluted with deionized water. Three readings and triplicate measurements were made for each sample.

#### 2.8.3 Particle size measurements

A Mastersizer 3000 lazer diffraction particle size analyzer (Malvern Panalytical, Shanghai, China) was used to evaluate the particle size of the Chia oil emulsions stabilized by OVA:AS polyelectrolyte complexes.

### 2.9 Spray drying of the chia oil emulsions stabilized with polyelectrolytes complexes of OVA:AS

Utilizing a lab-scale mini-spray dryer (Büchi, B-290, Switzerland) equipped with a 711 μm nozzle, the fine emulsions formed through high-pressure homogenization were introduced at a flow rate of 5 mL/min. The input air temperature was adjusted to 160 °C in accordance with earlier experiments. The resulting powdered dusts were collected for further analyses.

### 2.10 Characterization of chia seed oil microcapsules of emulsions stabilized by OVA:AS polyelectrolyte complexes

#### 2.10.1 Calculating the total oil contents

A slightly modified method described by K. Y. Li et al. (2022) was used to determine total oil contents (TOC) of powder of Chia oil microcapsules of emulsions stabilized by OVA:AS polyelectrolyte complexes. The capsule wall was ruptured by adding around 1 g (m1) of powder to 10 mL of 2 mol/L HCl solution and heating it in a water bath set at 90 °C for 30 min. After that, 10 mL of methanol and 40 mL of trichloromethane were added, and the mixture was vigorously spun to extract the oil. Following a 15-min centrifugation at 10,000 *g*, the organic phase was extracted, transferred onto an empty petri dish weighing m2, and dried at 60 °C in an oven until a consistent weight (m3) was achieved. Eq. 4 was then used to determine the powder's TOC:

(4) $$\mathrm{Total oil content}\;\left(\mathrm{TOC}\right)\%=\frac{m_{3}-m_{2}}{m_{1}}\times 100\;$$

#### 2.10.2 The calculation of the surface oil measurements

20 mL of petroleum ether was mixed with approximately 1 g (m_1_) of powder of Chia oil microcapsules of emulsions stabilized by OVA:AS polyelectrolyte complexes and gently stirred for 1 min. After filtration, the filtrate was washed once more using petroleum ether (10 mL). Then, the filtrates were combined and put into an empty petri plate weighing (m_2_). Then, a constant weight (m_3_) was obtained after drying it in an oven at 60 °C. Eq. 5 was used to determine the surface oil content (SOC), and Eq. 6 was used to determine the encapsulation efficiency (EE), respectively.

(5) $$\mathrm{Surface oil content}\;\left(\mathrm{SOC}\right)\%=\frac{m_{3}-m_{2}}{m_{1}}\times 100$$

(6) $$\mathrm{Encapsulation efficiency}\;\left(\mathrm{EE}\right)\%=\frac{\mathrm{TOC}-\mathrm{SOC}}{\mathrm{TOC}}\times 100\;$$

#### 2.10.3 Calculating the powder yield

Eq. 7 was used to calculate the yield of powder of Chia seed oil microcapsules of emulsions stabilized by OVA:AS polyelectrolyte complexes that was collected, weighed, and placed in the cyclone separator and spray dryer collector.

(7) $$\mathrm{Powder yield}\;\left(\%\right)=\frac{m_{1}}{m_{0}}\times 100$$

where m_1_ denotes the mass of the chia seed oil microcapsules that have been collected, and m_0_ signifies the overall solid content present in the emulsion.

#### 2.10.4 Measuring the moisture content

A consistent weight (m_2_) was achieved by placing around 1 g (m_1_) of the microcapsule powderin an oven set at 105 °C. Then Eq. 8 was used to determine the moisture content.

(8) $$\mathrm{Moisture content}\;\left(\%\right)=\frac{m_{1}-m_{2}}{m_{1}}\times 100$$

#### 2.10.5 Calculation of solubility

About 0.2 g (m_1_) powdered samples containing Chia seed oil microcapsules of emulsions stabilized by OVA:AS polyelectrolyte complexes were dissol*v*ed in distilled water (10 mL), and were filtered after magnetic stirring for 20 min. Weighing (m_2_) was done after the residue was collected and cooked to a consistent weight at 105 °C. Eq. 9 was used to determine the microcapsules' solubility.

(9) $$\mathrm{Solubility}\;\left(\%\right)=\frac{m_{1}-m_{2}}{m_{1}}\times 100$$

#### 2.10.6 Measurement of particle size

The powdered of Chia seed oil microcapsules of emulsions stabilized by OVA:AS polyelectrolyte complexes were determined using a Mastersizer 3000 lazer diffraction particle size analyzer (Mal*v*ern Panalytical, Shanghai, China).

### 2.11 Evaluation of the oxidation stability of chia seed oil microcapsules of emulsions stabilized by OVA:AS polyelectrolyte complexes

#### 2.11.1 Assessment of the oxidation stability

Chia seed oil's oxidation stability in emulsions stabilized by Chia oil microcapsules of emulsions stabilized by OVA:AS polyelectrolyte complexes was assessed using a slightly modified version of a prior study by K. Y. Li et al. (2022). Specifically, 5 mL of isooctane: isopropyl alcohol (1:2, *v*/v) was combined with 0.2 mL of emulsion, stirred for 1 min, then centrifuged at 3000 *g* for 3 min. 20 μL of the potassium thiocyanate solution (3.94 mol/L) and 20 μL of the FeCl_2_ solution (made by mixing 0.132 mol/L BaCl_2_ and 0.144 mol/L FeSO_4_ in the same volume ratio and filtering through a 0.22 μm filter) were then combined with 1 mL of the supernatant. After diluting the blend with the methanol: n-butanol mixture (1:2, v/v) to 5 mL, it was swirled for 20 s and allowed to sit at room temperature for 20 min in the dark. The absorbance (A) was subsequently measured at 510 nm. The control was made in the same way, but without the addition of emulsion. Eq. 10 was used to determine the POV (peroxide value).

(10) $$\mathrm{POV}\left(\mathrm{meq}/\mathrm{kg}\right)=\frac{0.5\times A\times k\times n}{2\times 55.86\times m}\times 1000$$

For this discussion, the letters A, n, k, and m in given equation denote the respective absorbance that was measured at 510 nm, the fraction of the supernatant volume, the slope of the Fe^3+^ standard curve (which is 2.82), and the mass of tuna oil that is present in the emulsion.

##### 2.11.1.1 Against temperature

A specific quantity of powder of Chia seed oil microcapsules of emulsions stabilized by OVA:AS polyelectrolyte complexes was kept in a dark, temperature-controlled chamber at 4 °C, 25 °C, and 50 °C, respectively, for 30 days. Then, using the procedure outlined in Section 2.11.1, POV determination was performed after around 1 g of powder was removed every 10 days.

##### 2.11.1.2 Against humidity

After 30 days in a dark, closed container with saturated solutions of KCl, Mg (NO_3_)^2^, or MgCl_2_, a specific quantity of powder of Chia seed oil microcapsules of emulsions stabilized by OVA:AS polyelectrolyte complexes for relative humidity levels of 40, 60, and 80 %, respectively. Then, using the procedure outlined in Section 2.11.1, around 1 g of powder was taken out every 10 days and submitted to POV determination.

##### 2.11.1.3 Against light exposure

A particular quantity of microcapsule powder was left in a sealed container exposed to either 24 h light, 24 h dark, and 12 h light/12 h dark at room temperature for 30 days. Then, using the procedure outlined in Section 2.11.1, POV determination was performed after around 1 g of powder was removed every 10 days.

### 2.12 Analysis of morphological features using scanning electron microscopy (SEM)

The surface morphology of the Chia seed oil microcapsules of emulsions stabilized by OVA:AS polyelectrolyte complexes powder was investigated using the ZEISS (Merlin Field Emission) SEM. Carbon tape was used to adhere the samples to metal stubs firmly. After that, a 45-s gold coating was applied to the samples. A resolution of 1.00 Kx and an accelerated voltage of 5 kV were used to take the micrographs. A magnification of 50-20 μm was used for the micrographs.

### 2.13 Statistical analyses

The data were presented as means ±SD, and each experiment was run in triplicate. A *t*-test was performed on the data using SPSS Statistics 18.0, and differences were considered significant at *p* < 0.05.

## 3 Results and discussion

### 3.1 Effect of pH

**Fig. 1:**
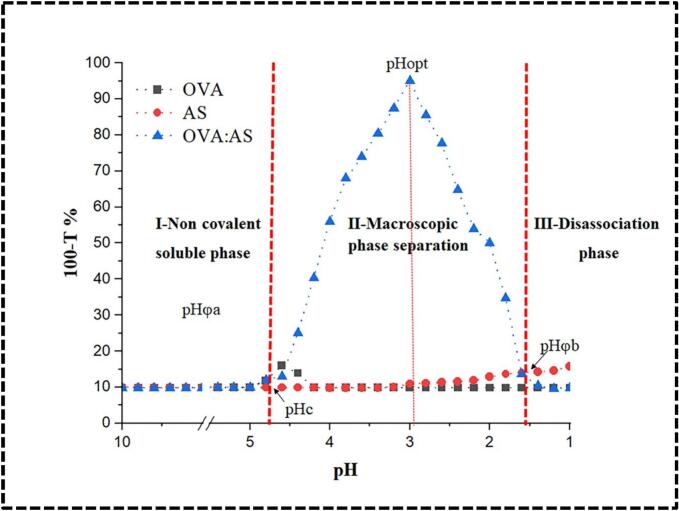
In a system with an OVA:AS ratio of 1:1, turbidity (100-%T) is influenced by pH levels, and the instability of complex coacervates is identified in regions labeled I, II, and III.

The formation of OVA:AS coacervate complexes (1:0, 0:1, 1:1) at 1–10 pH values revealed three different turbidity areas (I, II, III; Fig. 1A). The points where the two tangents intersected were identified as the transition zones (pHϕa, pHopt, pHϕb, and pHc). At pH levels close to the protein's isoelectric point (approximately 4.8 for ovalbumin), soluble complexes of polysaccharide and protein can be formed through non-covalent attractions between the polymers, according to turbidimetric titration and dynamic light scattering analyses conducted in region A (Souza & Garcia-Rojas, 2017). The complex formation, often known as pHc, causes a modest increase in turbidity at this pH. However, while titrating between pH 5.1 to 10, we did not observe any considerable variations in turbidity. Since the pH of the system was higher than the p*Ka* of starch (pKa 3.17), more deprotonation may have decreased the non-covalent interactions between the biopolymers, which might explain the lack of pHc behavior shown in area I. Turbidity rose at pH 4.88, close to ovalbumin's pI, signifying complex formation and aggregation as well as the start of macroscopic phase separation (pHϕa). Ovalbumin was protonated in area II as pH < pHϕa until the electrical charges between ovalbumin and AS were equal, and at pH 3.17 (pHopt), turbidity reached 95.3 %. The system became acidic, and AS stopped deprotonating at pH 3.17. Consequently, AS demonstrated the ability to interact with ovalbumin. Low turbidity readings in area III at pH 1.42 (pHϕb) suggested that the biopolymers had dissociated. Similar pH-dependent changes in the synthesis of ovalbumin were noted by Souza and Garcia-Rojas (2015) in the cases of pectin, egg white proteins, and xanthan gum, respectively. Furthermore, a minor rise in turbidity around its p*I* indicates that ovalbumin itself aggregated. Through electrostatic interactions with polysaccharides, protein aggregation may disrupt charged residues or charge patches of proteins, even though these tiny aggregates did not seem to stop interacting with ovalbumin and AS (Y. Li et al., 2014).

### 3.2 The impact of OVA to AS ratio

**Fig. 2:**
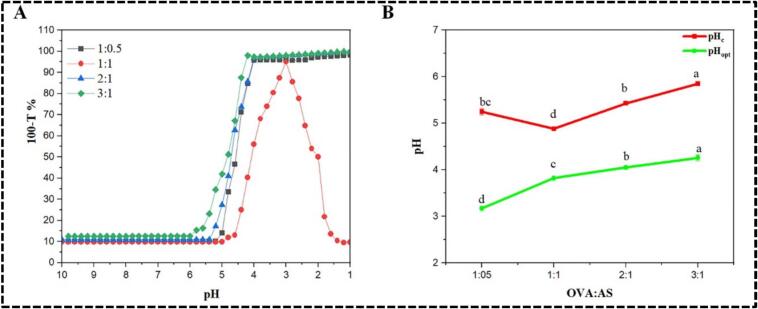
(A) Relationship between turbidity (100-T%) and pH in systems with varying mass ratios of OVA:AS. (B) Changes in the pHc and pHopt of the complexes formed with different mass ratios of OVA:AS.

The protein-polysaccharide ratio impacted the charge balance, the level of self-aggregation, and the strength of contacts during complexation (Gu et al., 2024). Consequently, the effect of the OVA:AS ratio on complex coacervation formation was examined in the absence of salt ions. When the OVA:AS ratio was increased from 1:0.5 to 3:1 at a fixed concentration of total biopolymer (0.1 %, *w*/w), the turbidity improved, as shown in Fig. 2A. At larger mixing ratios, changes in pHc and pHopt also demonstrate the somewhat greater contribution of turbidity from OVA aggregates during complex formation. Protein molecules attach to polysaccharide chains, which encourages protein aggregation until a plateau is achieved. However, proteins may pack together to form aggregates when there is an excess of protein. The progressive inhibition of biopolymer dissociation was also brought on by this aggregation (Souza & Garcia-Rojas, 2015). Fig. 2B illustrates the variations in critical pH (pHc and pHopt) of mixtures as a function of OVA: AS ratios. As the OVA ratio rose in 1:0.5, 2:1, and 3:1, the pHc and pHopt rose as well. The quantity of OVA molecules accessible per AS chain significantly influenced the development of the electrostatic complex. As seen in Fig .2B, the key pH transition sites of the OVA: AS biopolymer ratio directly impacted the complexes. The pHc and pHopt were moved to lower pHs by the increase in protein. Phase separation brought on by the aggregation of the soluble complexes might account for the reliance of pHc on the OVA/AS ratio. This happened as a result of the growing quantity of positively charged ovalbumin molecules, which encourages the early neutralization of negative carboxyl groups (COOH) in starch. Previous reports indicate that critical pH values are dependent on the mixing ratio. In this instance, as the ratios increased, the critical pH values changed before plateauing. According to the investigators, this phenomenon happens because more protein molecules are accessible for binding per polysaccharide chain (Xiong et al., 2016).

### 3.3 Fourier-transform infrared spectroscopic (FTIR) analysis

**Fig. 3:**
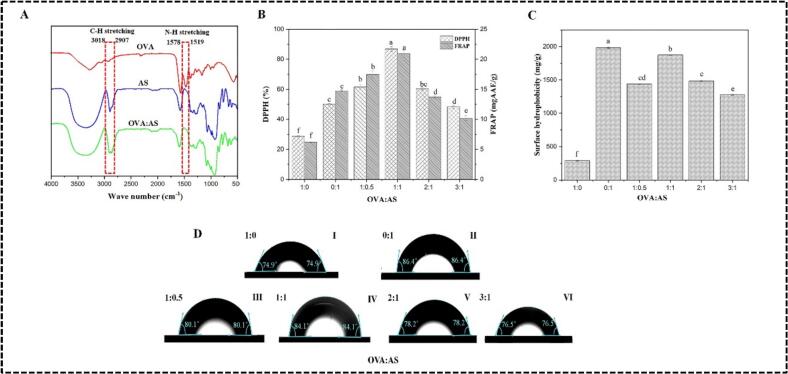
Effect of OVA to AS mass ratio polyelectrolyte complexes on: (A): FTIR spectrum; (B): DPPH free radical scavenging rate and ferric reducing antioxidant power; (C): The surface hydrophobicities measure by 8-Anilino-1-naphthalenesulfonic acid (ANS) binding; (D): The Contact angel (θ). Values without same letter(s) are significantly different *p* < 0.05.

The interactions between the functional groups of the polyelectrolytes' constituents (OVA (1:0), AS (0:1), OVA:AS (1:1)), and chemical bonding functionality, as well as modifications to hydrogen bonds and protein secondary structure, may all be determined using the FTIR spectra of the microcapsules seen in Fig. 3A (Cao et al., 2024). OVA displayed absorption bands associated with N—H stretching at 1519 cm-1 (amide II) and 1578 cm-1 (amide I), as well as C=O stretching at 1425 cm-1 (free carboxyl group), as shown in Fig. 3A. The proteins' strongest amide I and II bands, which ranged from around 1578 to 1480 cm-1, respectively, were their most distinguishing spectral properties (Souza & Garcia-Rojas, 2017). The amide III (-NH 3+ groups) and the C—O stretching vibration were linked to the peaks in AS at 1551 cm-1 and 1030, respectively (Muhammad et al., 2022). After complexation, the OVA:AS polyelectrolyte complex (1:1) exhibited a distinctive peak at 1519 cm-1 to 1578 shifts due to NH stretching and at 2907 to 3018 cm-1 shifts due to CH bond stretching. This suggests that the carboxyl and amine groups of OVA and AS conducted electrostatic interactions. Furthermore, a wide band at around 3000–3600 cm-1 was seen in all of the OVA:AS (1:1) complexes, suggesting that hydrogen bonding played a role in the complexes' development as well. The consistency of this behavior can also be observed during the coacervation between soybean protein isolate and chitosan (Cao et al., 2024).

### 3.4 Antioxidant properties

The polyelectrolytes complexes of OVA:AS were evaluated for their antioxidant capabilities. With regard to DPPH free radical scavenging activity and FRAP, AS demonstrated 1.75- and 2.37-times greater levels than OVA, as can be observed in Fig. 3B. Following their affiliation, there was a notable increase in activity. The highest values were found at 86.79 ± 1.61 % (DPPH) and 20.91 ± 0.58 mg AAE/g (FRAP), at a mass ratio (OVA: AS) of 1:1, which was 1.72 and 1.41 times that of AS. The DPPH free radical scavenging activity decreased significantly to 60.41 ± 0.85 % and 48.35 ± 1.04 %, respectively, as the OVA ratio increased to 2:1 and 3:1, and the FRAP scavenging activity decreased slightly to 13.72 ± 0.21 mg AAE/g and 10.18 ± 0.44AAE/g, respectively. These differences supported the theory that AS was primarily responsible for the complexes' antioxidant activity. The groups of hydroxyl in AS and free groups of active amino acids and mercaptan in OVA contribute to their antioxidant properties (Ma et al., 2019). More active groups may have been exposed as a result of the interactions and structural rearrangement of the polyelectrolytes constituents, giving the complexes increased antioxidant activity.

### 3.5 Surface hydrophobic characteristics of the OVA/AS polyelectrolyte complexes

As a hydrophobic fluorescence probe, 8-Anilino-1-naphthalenesulfonic acid (ANS) is often employed to investigate conformational changes and the surface exposure of protein hydrophobic regions (Qiu et al., 2017). Fig. 3C illustrates that the surface hydrophobicity of O*V*A:AS polyelectrolyte complexes at ratios of 1:0.5, 1:1, 2:1, and 3:1 exceeded that of the 1:0 ratio (OVA:AS), measured at 288.67 ± 6.06 μg/g, while remaining lower than the 0:1 ratio (OVA:AS), which was 1980 ± 8.78 μg/g. There was a rise in hydrophobicity, measuring 1438 ± 6.53 μg/g and 1873 ± 5.44 μg/g, respectively, when the AS ratio increased from 1:0.5 to 1:1. The findings suggested that ANS was capable of binding to the cation present on the surface of OVA, resulting in a structural alteration of OVA following the complexation with ANS. This phenomenon led to the exposure of the OVA's hydrophobic groups on the molecular surface. These findings are aligned with the findings as reported by Dan et al. (2019). With the increase in the OVA ratio to 2:1 and 3:1, there was a notable decline in binding ability, measured at 1484 ± 9.74 μg/g and 1276 ± 7.62 μg/g, respectively (Fig. 3C). These findings suggested that OVA underwent a significant structural change as a consequence of the interaction with AS, exposing more hydrophobic residues. This might help to explain the differences in the antioxidant characteristics seen in Fig. 3B and the contact angle shown in Fig. 3D. This suggests that the complexes' wettability was significantly influenced by their composition, and that a high AS% preferred this characteristic, which was advantageous for creating Pickering emulsions with strong antioxidant activity.

### 3.6 Contact angle analysis of the OVA: AS polyelectrolyte complexes

The capacity of particles to stabilize Pickering emulsions may be demonstrated by their angle of contact (θ) (Muhammad, Ramzan, Abdullah Abbas, et al., 2025). The contact angle (Fig. 3D (I, II, III, IV, V, VI) of OVA (1:0) was 74.9° (Fig. 3DI), which was quite similar to the value in a prior publication (Qiu et al., 2017). Whereas, as shown in Fig. 3DII), the contact angle of AS (0:1) was near to 86.4°. In comparison to AS alone, the contact angle decreased after the two polyelectrolytes complexed. The contact angles for assemblies in mass ratios (OVA:AS) 1:0.5 and 1:1 were 80.2° (Fig. 3DIII) and 84.1° (Fig. 3DIV), respectively. With the increasing ratio of OVA, there was a significant decline in the contact angle, dropping below 80°, specifically to 78.2° at a 2:1 ratio (Fig. 3DV) and 76.5° at a 3:1 ratio (Fig. 3DVI).

### 3.7 Analysis of the emulsifying properties

The emulsifying stability index (ESI) and emulsifying activity index (EAI) serve as essential indicators for evaluating the emulsifying characteristics of proteins. EAI primarily measures how well a protein can attach to the surface of oil droplets and create emulsions with both water and oil. On the other hand, ESI assesses the stability of oil droplets dispersed in the emulsion over time, as well as their ability to resist coalescence and sedimentation (Sun et al., 2023). Table 1 presents the EAIs and ESIs for OVA:AS. As the ratio of AS to OVA increased, the EAIs and ESIs also rose, reaching 41.5 ± 0.08 m2/g and 66.1 ± 0.05 min for a 1:0.5 ratio, and 60.9 ± 0.03 m2/g and 84.2 ± 0.08 min for a 1:1 ratio. Conversely, a decline was noted when the OVA to AS ratio increased from 2:1 to 3:1, indicating that the emulsifying properties of OVA were enhanced through interaction with the starch particles of AS. The complexation of OVA with AS expanded the adsorption area at the oil-water interface, thereby boosting the EAIs.

**Table 1:** Emulsification properties of different OVA to AS mass ratio polyelectrolyte complexes.

OVA:AS ratio	Emulsifying activity index (m^**2**^/g)	Emulsifying stability index (min)
1:0	18.2 ± 0.04^f^	27.6 ± 0.07^f^
0:1	28.6 ± 0.02^e^	39.5 ± 0.09^e^
1:0.5	41.5 ± 0.08^c^	66.1 ± 0.05^c^
1:1	60.9 ± 0.03^a^	84.2 ± 0.08^a^
2:1	44.7 ± 0.05^b^	70.7 ± 0.04^b^
3:1	35.1 ± 0.06^d^	58.3 ± 0.06^d^

There are significant differences (*p* ≤ 0.05) in the mean ± standard deviation values for each solution in the same column, denoted by different superscripts.

The observed increase in the electrostatic stability indices (ESIs) can be attributed to the enhanced electrostatic repulsion among emulsion droplets upon the addition of AS. Furthermore, as shown in Fig. 3C, the observed increase in surface hydrophobicity suggests more effective emulsification of ovalbumin (OVA) in the presence of AS. In AS starch, the incorporation of hydrophobic groups converts starch granules into amphiphilic macromolecules, improving their surface activity at the oil/water (O/W) interface (Ji et al., 2022). The enhanced emulsifying properties of OVA can be attributed to changes in the protein structure, which expose more hydrophobic regions. As a result of the interactions between starch and OVA, the attraction of the protein to the oil–water interface was enhanced (Chen et al., 2024).

### 3.8 Microscopic and visual illustration of chia oil emulsions stabilized by OVA:AS polyelectrolyte complexes before and after HPH

The preparation of emulsions on an industrial scale for spray drying often involves a method known as high-speed dispersion, which is then followed by high-pressure homogenization (HPH). A high-pressure pump and a homogenizing nozzle are the two different components that make up this method. In order to achieve a fine emulsion at the output of the homogenizer, a step of pre-emulsification is advised to generate a primary coarse emulsion prior to the subsequent phase. This pre-emulsification stage is often carried out via a technique known as high-speed dispersion methodology. It is common practice in the food business to use homogenization for the purpose of stabilizing emulsions by avoiding creaming and coalescence, reducing particle size (dispersion), and mixing in components (Hu et al., 2023).

**Fig. 4:**
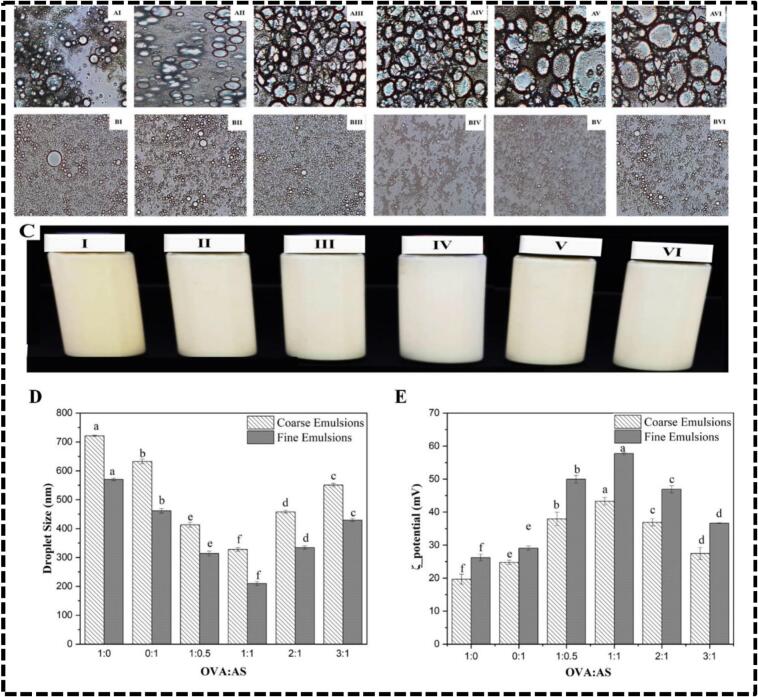
The Microstructures of Chia oil conventional and Pickering emulsions stabilized by OVA: AS complexes: (A): fine emulsion; (B): Coarse emulsion; (C): Represents the visual appearance of Chia oil conventional 1:0 (I), 0:1 (II), and Pickering emulsions 1:0.5 (III), 1:1 (IV), 2:1 (V), 3:1 (VI), stabilized by OVA: AS complexes; (D): droplet size; (E): zeta potential of the coarse and fine Chia oil conventional (1:0, 0:1) and Pickering emulsions (1:0.5, 1:1, 1:1, 2:1, and 3:1) stabilized by OVA: AS complexes. Values marked with different letters indicate a significant difference (p < 0.05), and comparisons were made between the values within the same column.

To date, only a limited number of Pickering emulsions have undergone this treatment. Chia oil conventional and Pickering emulsions were created in this study utilizing industrial processes using OVA/AS complexes. The microscopic illustration of coarse (Fig. 4A) and fine (Fig. 4B) emulsions and the visual look of the fine emulsion after HPH treatment also given in Fig. 4C. The zeta potentials and droplet sizes of coarse and fine emulsions were presented (Fig. 4D, E, respectively). There were two possible explanations for the dramatic reduction in particle size after HPH, given that the fine emulsions were noticeably smaller than their coarse counterparts: (1) During the reassembly of droplets, more soluble proteins and phospholipids could be adsorbed to the interface, preventing oil droplet accumulation; (2) Protein aggregates could undergo structural changes that make them more flexible, leading to increased protein-protein interactions and improved emulsifying capacity. The reduction in particle size corresponds to the rise of particular surface area, which might also enhance the adsorbing capacity of protein; the smaller size supports greater emulsion stability and higher emulsion activity (Liu et al., 2023).

### 3.9 Zeta potential and droplet size of the chia oil emulsions stabilized by OVA:AS polyelectrolyte complexes

Overall, for both coarse and fine emulsions, the droplet size was found to be 1:0 > 0:1 > 3:1 > 2:1 > 1:0.5 > 1:1. The OVA to AS mass ratio of 1:1 resulted in the smallest droplet size, as shown in Fig. 4D, dropping from 327.86 ± 6.40 nm (coarse) to 209.30 ± 7.00 nm (fine). According to our hydrophobicity result (Fig. 3C), the protein's unfolding revealed a hydrophobic group that might function as an emulsifier. 1:1 showed the maximum hydrophobicity (Yan et al., 2017). The fine emulsions showed a considerable rise in zeta potential in contrast to droplet size (Fig. 4D). Zeta potential may evaluate the stability of the respective emulsion to a certain degree. In order to prevent aggregation and enhance the stability of the emulsion, the increase in charge will strengthen the repulsive force between the droplets. The increase of hydrophobic groups on the protein and reconfiguration of its molecular structure might be due to the HPH treatment. Overall, it was found that the zeta potential for both coarse and fine emulsions was 1:0 < 0:1 < 3:1 < 2:1 < 1:0.5 < 1:1. According to the data shown in Fig. 4E, the fine and coarse emulsions stabilized with the OVA/AS complexes built in a mass ratio of 1:1 had the largest zeta potentials, measuring 43.28 ± 1.12 mV and 57.70 ± 0.36 mV, respectively. Having a zeta potential of more than 30 mV was advantageous for their stability in further processing (Huo et al., 2019).

### 3.10 The performance of spray drying for chia oil microcapsules of emulsions stabilized by OVA: AS polyelectrolyte complexes

**Fig. 5:**
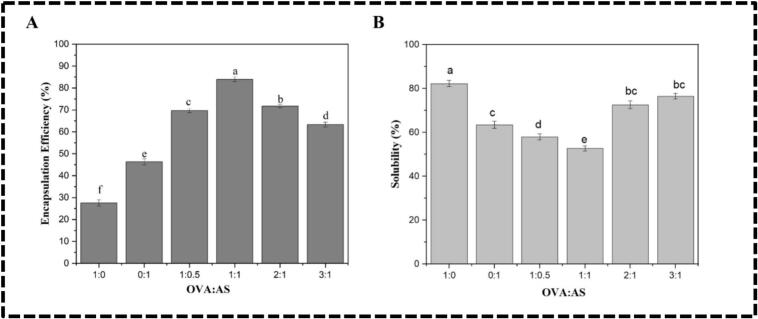
(A): Encapsulation Efficiency; (B): Solubility of Chia oil microcapsules derived from conventional (1:0, 0:1) and Pickering emulsions (1:0.5, 1:1, 1:1, 2:1, and 3:1) stabilized by OVA:AS polyelectrolyte complexes. Values marked with different letters indicate a significant difference (p < 0.05), and comparisons were made between the values within the same column.

Spray drying was applied to the chia oil emulsions stabilized by OVA: AS polyelectrolyte complexes; the powder characteristics were compiled. The important physicochemical characteristics shown in Table 2 are powder yield, surface oil, moisture content, and total oil. The quality of the encapsulated powders was assessed by means of encapsulation efficiency (Fig. 5A), and solubility (Fig. 5B). Table 2 illustrates that the spray-dried powder of chia oil microcapsules loaded emulsions stabilized by OVA:AS polyelectrolyte complexes achieved a total oil content ranging from 52.73 ± 0.93 % to 68.90 ± 0.50 %, which is in proximity to the theoretical value of 70 %. This suggests that the emulsion may be utilized to create powders with exceptionally high oil content. This value significantly exceeded the 39.23 ± 0.27 % observed for OVA alone (1:0) stabilized microcapsules. Spray-dried powder quality, such as storage dispersibility, flowability, and oxidative stability, is greatly influenced by the surface oil concentration. In contrast, the powder yield greatly influences the economics of a spray drying process. The emulsions demonstrated increased effectiveness in hindering oil leakage, and SOC reduced in microcapsules as the contents of AS rose such as 1:0.5 (17.38 ± 1.56 %) and 1:1 (11.06 ± 1.37 %) in polyelectrolyte complexes, in contrast to the dried conventional emulsion (1:0), which exhibited the highest value at 28.42 ± 1.23 %. The highest encapsulation efficiency recorded was 83.94 ± 1.05 % (Fig. 5A). Similar variations in powder yield were observed with surface oil content; the highest yield (77.09 ± 1.13 %; Table 2) was found in chia oil microcapsules of OVA:AS mass ratio of 1:1, while the lowest yield (49.71 ± 1.87 %) was found in the chia oil microcapsules of OVA-stabilized conventional emulsions (1:0). This observation implies that the leaked oil caused the powders to cling to the interior of the dryer chamber. Generally speaking, both variants supported the idea that raising the input air temperature improves the spray drying process's microencapsulation efficiency. However, too much heat would accelerate the evaporation of water, resulting in surface fissures in the microcapsule and, ultimately, oil leakage (K. Y. Li et al., 2022). Without any notable variations, the moisture levels of all the polyelectrolyte complexes, chia oil-loaded emulsion powders, were less than 4 %, which was enough to ensure their shelf life (S. Wang et al., 2018).

**Table 2:** Spray-dried powder characteristics such as total oil content, surface oil content, moisture content, and powder yield of Chia oil microcapsules derived from conventional (1:0, 0:1) and Pickering emulsions (1:0.5, 1:1, 1:1, 2:1, and 3:1) stabilized by OVA: AS polyelectrolyte complexes.

OVA:AS ratio	Total oil content (%)	Surface oil content (%)	Moisture content (%)	Powder yield (%)
1:0	39.23 ± 0.27^f^	28.42 ± 1.23^a^	1.87 ± 0.35^a^	49.71 ± 1.87^e^
0:1	45.84 ± 0.34^e^	24.61 ± 1.09^b^	2.08 ± 0.21^a^	57.19 ± 1.09^d^
1:0.5	57.25 ± 0.56^c^	17.38 ± 1.56^cd^	2.34 ± 0.27^a^	69.25 ± 1.56^b^
1:1	68.90 ± 0.50^a^	11.06 ± 1.37^e^	2.41 ± 0.19^a^	77.09 ± 1.13^a^
2:1	59.08 ± 0.82^b^	16.70 ± 0.07^d^	2.30 ± 0.30^a^	68.54 ± 1.79^b^
3:1	52.73 ± 0.93^d^	19.36 ± 0.24^c^	2.23 ± 0.10^a^	61.34 ± 1.44^c^

There are significant differences (p ≤ 0.05) in the mean ± standard deviation values for each solution in the same column, denoted by different superscripts.

Chia oil microcapsules of emulsions stabilized by O*V*A:AS polyelectrolyte complex at a 1:1 ratio had the highest moisture content at 2.41 ± 0.19 % in comparison to the ratios 1:0, 0:1, 1:0.5, 2:1, and 3:1 (OVA:AS). Table 2 demonstrates that the OVA:AS combination had greater effectiveness for these parameters relative to OVA. The polyelectrolyte complex Pickering emulsion showed superior efficacy in preventing oil leakage relative to the conventional emulsion (1:0 (OVA), 0:1 (AS)). Additional measures, like the modification of emulsion surface properties or the integration of drying agents into the emulsion, should be undertaken to reduce surface oil content and improve powder yield. So far, a restricted number of researchers have investigated the spray drying parameters of polyelectrolyte complex Pickering emulsions. According to (K. Y. Li et al., 2022), the ovalbumin-Gum Arabic polyelectrolyte complex-stabilized Perilla seed oil Pickering emulsion could be spray-dried to create powders with an oil content of 74.68 %. However, the powder yield was only 37.32 %. In the presence of maltodextrin, another study found that the spray-dried alginate-coated chitosan-stabilized Pickering emulsion only produced around 21 % powder (Singh et al., 2021).

The microcapsules exhibited significant differences in solubility. As shown in Fig. 5B, as the percentage of AS in the complex increased, the parameter decreased. For example, the microcapsules made from the OVA-stabilized emulsion (1:0) reached a maximum of 82.31 ± 1.47 %, whereas the emulsion stabilized by the OVA:AS complexes assembled in a mass ratio of 1:1 only reached 52.77 ± 1.25 %. This may pertain to their varying wettability, as seen in Fig. 3D; as wettability rises, solubility reduces. Therefore, the present study showed that the Pickering emulsions of chia oil based on the OVA: AS polyelectrolyte complex had extra benefits as an emulsifier for spray drying.

### 3.11 Investigation of the morphology of chia oil microcapsules of emulsions stabilized with OVA:AS polyelectrolyte complexes

**Fig. 6:**
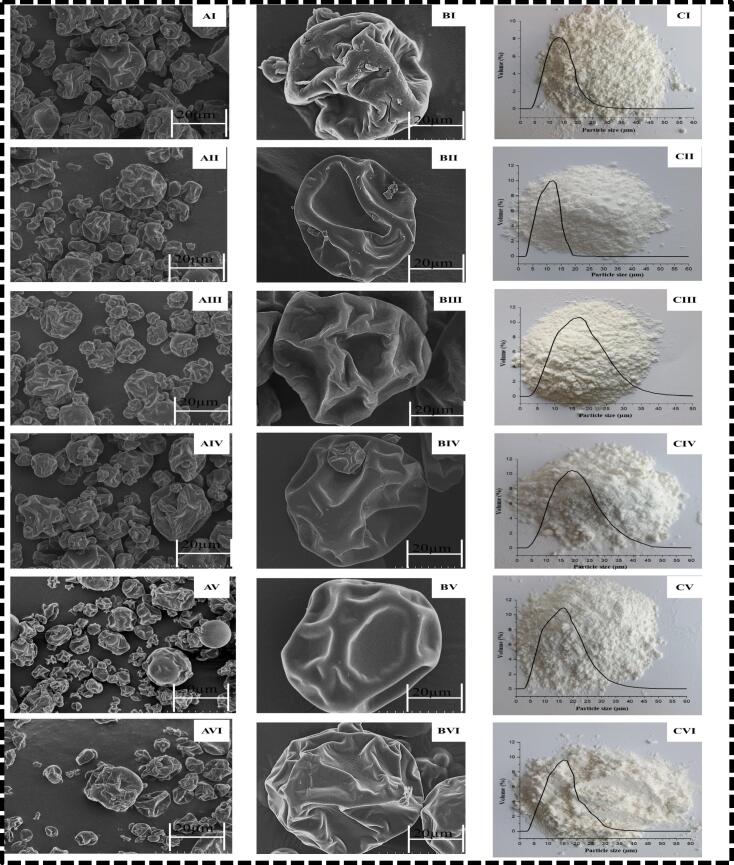
SEM images of chia seed oil microcapsules formed from both conventional and Pickering emulsions, which are stabilized by OVA:AS polyelectrolyte complexes at varying mass ratios of 1:0 (I), 0:1 (II), 1:0.5 (III), 1:1 (IV), 2:1 (V), and 3:1 (VI), are shown at magnifications of 2000× (A) and 10,000× (B); (C) Particle size distribution and appearances of the spray dried Chia seed oil microcapsules derived from conventional 1:0 (I), 0:1 (II) and Pickering emulsions 1:0.5 (III), 1:1 (IV), 2:1 (V), 3:1 (VI) stabilized by OVA: AS polyelectrolyte complexes.

The micrographs of chia oil microcapsules obtained from scanning electron microscopy, depicted in Fig. 6 A, B (I, II, III, IV, V, VI), illustrate that the microcapsules exhibited a more spherical shape and smoother surface, with fewer dents and reduced shrinkage. Moreover, significant aggregation was observed in the microcapsules formed from the conventional emulsions stabilized with OVA (1:0), as shown in Fig. 6 AI, compared to those from AS-based (0:1) polyelectrolyte complexes, suggesting that OVA is less effective as a wall material for encapsulating chia oil. This was further supported by the low encapsulation efficiency of this sample (Fig. 5A). Nevertheless, the microcapsules formed from the four chia oil microcapsules, which were loaded with emulsions stabilized by OVA: AS polyelectrolyte complexes in ratios of 1:0.5, 1:1, 2:1, and 3:1, exhibited more intact structures and less aggregation (Fig. 6 A, B (III-VI)). This is a typical trait of spray-dried microcapsules. The results are consistent with those found in microcapsules of perilla seed oil, which were created from a Pickering emulsion stabilized by a polyelectrolyte complex of OVA and gum Arabic (K. Y. Li et al., 2022). Moreover, Fig. 6 C I-VI illustrates the appearance of the six chia oil microcapsules. The image reveals that creamy white powders were formed, indicating the successful encapsulation of the yellow chia oil within the microcapsules. Notably, the powder derived from the OVA-stabilized conventional emulsion (1:0) displayed a darker coloration, which can be attributed to its elevated surface oil content, as indicated in Table 1.

It has been suggested that oil powders related to emulsions with greater interfacial elasticity have lower aggregation and a smoother surface. Polyelectrolyte complexes exhibit significant viscoelasticity and are capable of forming continuous coatings around oil droplets (Gadkari et al., 2019). Therefore, the microcapsules produced by using protein/modified polysaccharide polyelectrolyte complexes possess improved morphological features pertaining to their enhanced viscoelastic properties. Figs. 6A, B I–VI also showed that the microcapsules made from the Pickering emulsions had dents and shrinkage. However, the chia oil microcapsule loaded by emulsions stabilized by OVA: AS polyelectrolyte complexes assembled in a mass ratio of 1:1 (Fig. 6A, B IV) offered significantly improved structural integrity, as shown by the absence of empty microcapsules, much smaller dents, and less shrinkage.

However, the six microcapsules showed no significant variation in particle size, with measurements of 13.09 μm (1:0), 12.24 μm (0:1), 17.46 μm (1:0.5), 18.92 μm (1:1), 16.70 μm (2:1), and 15.39 μm (3:1), respectively (Fig. 6C). This might be connected to the improved spray drying capabilities, as can be seen in Table 2.

### 3.12 Assessment of the oxidation constancy of chia seed oil and its microcapsules

Reducing oxidation and shielding chia oil from the damaging effects of external stressors, including humidity, temperature, and light, are the main goals of microencapsulating the oil. These stresses intensify oxidation, which causes oils to become rancid. The kind of processing techniques and wall materials used throughout the process of microencapsulation could determine the effectiveness of the encapsulation. The processes, such as cross-linking, emulsification, and drying, significantly impact the effectiveness of microencapsulation (Timilsena et al., 2016).

#### 3.12.1 Stability against temperature

**Fig. 7:**
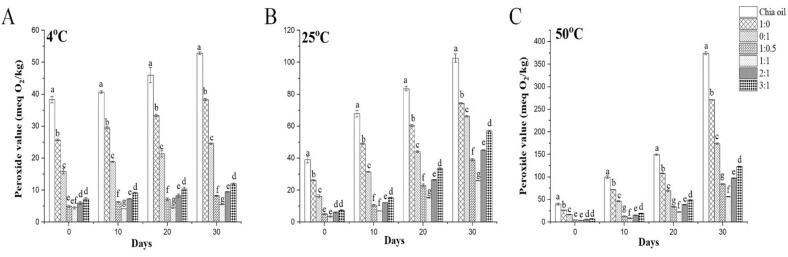
This figure illustrates the peroxide levels in chia oil and the spray-dried microcapsules. These were created using both regular (1:0, 0:1) and Pickering emulsions (1:0.5, 1:1, 1:1, 2:1, and 3:1) with OVA: AS polyelectrolyte complexes. The study monitored these levels over 30 days at three temperatures: 4 °C (A), 25 °C (B), and 50 °C (C). Values marked with different letters indicate a significant difference (p < 0.05), and comparisons were made between the values within the same column.

Tests of storage stability, which are conducted at various temperatures, provide an accurate picture of oxidative stability. Chia oil and its OVA:AS complexes stabilized microcapsule were therefore additionally subjected to oxidative stability tests by storing at three temperatures, 4, 25, and 50 °C, for 30 days, with assessments conducted at 10-day intervals (0,10,20,30). The oxidation that has taken place during storage was shown by the measurement of the peroxide values (POV). As the temperature rose and the time elongated, all of the microcapsules' oxidation increased, as seen in Figs. 7A, B, and C. The oxidation stability of emulsions was significantly enhanced by entrapment, and the Pickering emulsions performed effectively in this respect.

The POVs of free chia oil were recorded as 52.92 ± 0.38 meqO_2_/kg, 102.69 ± 2.59 meqO_2_/kg, and 374.32 ± 3.21 meqO_2_/kg, respectively, after 30 days of storage at 4 °C, 25 ± C, and 50 °C. In contrast, the POVs of the microcapsules prepared from the chia oil microcapsule loaded in OVA:AS polyelectrolyte complexes-stabilized emulsions, assembled in a mass ratio of 1:1, decreased by 5.47 ± 0.04 meqO_2_/kg, 26.01 ± 0.13 meqO_2_/kg, and 56.27 ± 0.68 meqO_2_/kg, respectively. This was more effective than OVA (38.36 ± 0.47 meqO_2_/kg, 74.45 ± 0.59 meqO_2_/kg, and 271.25 ± 0.50 meqO_2_/kg, respectively), and the OVA: AS complexes gathered in mass ratios 1:0.5, 2:1, and 3:1 also displayed a comparable pattern of POV for increased temperatures, as it can be seen in (Fig. 7A, B, C). These findings were aligned with those shown in Fig. 3B, as well as another study that found that an increase in the amount of chitosan hydrochloride in the wall material improved the macadamia oil's capacity to be stored in microcapsules (Zhong et al., 2021). Likewise, it has also been observed that the interactions of chia seed gum complex coacervates and chia seed protein isolate created a barrier that protects chia seed oil from oxidation at elevated temperatures (Timilsena et al., 2016).

#### 3.12.2 Stability against humidity

**Fig. 8:**
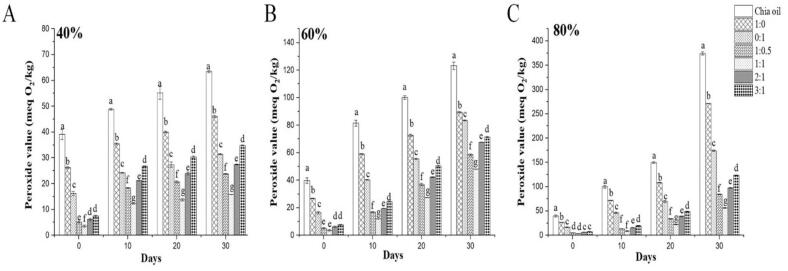
Peroxide values of Chia oil and its spray dried microcapsules derived from conventional (1:0, 0:1) and Pickering emulsions (1:0.5, 1:1, 1:1, 2:1, and 3:1) stabilized by OVA: AS polyelectrolyte complexes during 30 days of storage at different relative humidity: 40 % (A); 60 % (B); and 80 % (C). Values marked with different letters indicate a significant difference (p < 0.05), and comparisons were made between the values within the same column.

It has been noted that chia oil is sensitive to ambient relative humidity (RH), and that RH has been utilized in food products to regulate the lipid oxidation (Timilsena et al., 2016). Therefore, the stability of Chia oil and its OVA:AS complexes stabilized microcapsules under varying humidity levels was also examined. After being stored in RHs 40 % (A), 60 % (B), and 80 % (C) for 30 days, as shown in Fig. 8 A, B C, respectively. A high RH significantly increased the oxidation of free chia oil and its POV, rising from the initial values of 63.50 ± 0.40 meqO_2_/kg, 123.23 ± 1.69 meqO_2_/kg, and 419.24 ± 3.39 meqO_2_/kg, respectively. Since every microcapsule showed the same level of sensitivity to RH, it is possible that the composition of the microcapsule walls may have an impact on chia oil's sensitivity against RH. The OVA: AS mass ratio of 1:1 showed the maximum efficiency, and the microcapsules offered sufficient protection against a range of RHs. The microcapsules' POVs increased to 15.87 ± 0.03 meqO_2_/kg, 48.11 ± 0.13 meqO_2_/kg, and 84.37 ± 0.68 meqO_2_/kg after 30 days of storage in the three RHs. This was significantly less than the microcapsules produced in the mass ratios of 1:0.5, 2:1, and 3:1, as well as those stabilized by 1:0 (OVA) of 46.03 ± 0.38 meqO_2_/kg, 89.33 ± 0.50 meqO_2_/kg, and 303.91 ± 0.51 meqO_2_/kg. These findings showed that chia oil was effectively shielded against accelerated oxidation in a range of RHs by microencapsulation via the OVA: AS complexes as emulsion stabilizers. This may be attributed to the varying interfacial characteristics of the stabilizers, as illustrated in Fig. 3C, where increased hydrophobicity enhanced the repulsion of water from the environment (Hoang et al., 2021), thereby delaying chia oils' oxidation within the microcapsules.

#### 3.12.3 Assessment of stability against exposure to light and dark

**Fig. 9:**
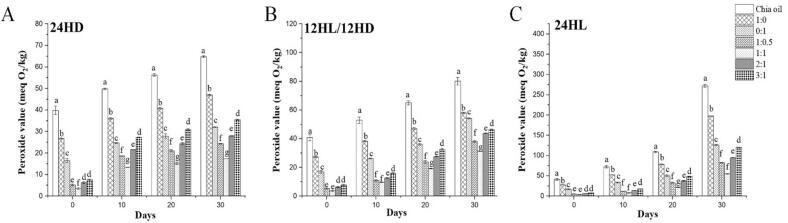
Peroxide values of Chia oil and its spray dried microcapsules derived from conventional (1:0, 0:1) and Pickering emulsions (1:0.5, 1:1, 1:1, 2:1, and 3:1) stabilized by OVA: AS polyelectrolyte complexes during 30 days of storage after exposure to 24 h dark period (A); 12 h light and 12 h dark period (B); 24 h light period (C). Values marked with different letters indicate a significant difference (p < 0.05), and comparisons were made between the values within the same column.

Following an exposure to light (L) and dark (D), the oxidation stability of free chia oil and its OVA:AS complexes stabilized microcapsule powder was examined. Fig. 9 A, B, and C show 24 h L,12 h L/12 h D and 24 h L for 30 days. It is evident that when the storage duration was extended, the POVs of every sample increased. The POV of the free chia oil, which was light-sensitive, was 272.50 ± 3.40 meqO_2_/kg as opposed to 94.77 ± 0.39 meqO_2_/kg after 30 days of dark storage. However, the microcapsules appeared to exhibit reduced sensitivity after exposing to the light. After 30 days of light exposure, the POVs of the studied microcapsules were 197.54 ± 0.52 meqO_2_/kg (1:0) and 126.65 ± 1.48 meqO_2_/kg (0:1), compared to 82.38 ± 0.59 meqO_2_/kg (1:0.5), 54.84 ± 0.71 meqO_2_/kg (1:1), 95.06 ± 0.69 meqO_2_/kg (2:1), and 120.41 ± 0.63 meqO_2_/kg (3:1). In contrast, the values after 30 days in the dark were 46.95 ± 0.39 meqO_2_/kg (1:0) and 32.07 ± 0.18 meqO_2_/kg (0:1), compared to 24.32 ± 0.13 meqO_2_/kg (1:0.5), 17.46 ± 0.03 meqO_2_/kg (1:1), 28.60 ± 0.11 meqO_2_/kg (2:1), and 35.54 ± 0.24 meqO_2_/kg (3:1), indicating that the microcapsule shell effectively shielded against light exposure (Elkalla et al., 2023).

## 4 Conclusions

Pickering emulsions stabilized by high antioxidant-based OVA/AS polyelectrolyte complexes serve as promising feeds for producing oxidation-stable PUFA microcapsules through spray drying. OVA has the potential to influence AS significantly, enhancing its hydrophobicity, wettability, and emulsifying characteristics. The elevated percentage of AS in the complex promotes stability by improving the emulsions' total solid concentration and reducing the microcapsules' surface oil content. The optimal stability was achieved in OVA/AS with a mass ratio of 1:1, yielding POVs of 56.27 ± 0.68 meqO_2_/kg at 50 °C, 84.37 ± 0.68 meqO_2_/kg at 80 %, and 54.84 ± 0.71 meqO_2_/kg in light. In contrast, the microcapsules derived from the OVA-stabilized conventional emulsion microcapsules (1:0; OVA/AS) exhibited values of 271.25 ± 0.50 meqO_2_/kg at 50 °C, 303.91 ± 0.51 meqO_2_/kg at 80 %, and 197.54 ± 0.52 meqO2/kg in light for 30 days. These microcapsules may be utilized in the food industry to produce instant beverages, seasonings, and a range of functional foods rich in PUFA content. Our research offered a fresh perspective on using OVA-NL within the food industry.

## CRediT authorship contribution statement

**Rabia Ramzan:** Writing – original draft, Visualization, Validation, Software, Resources, Methodology, Investigation, Funding acquisition, Formal analysis, Data curation, Conceptualization. **Zafarullah Muhammad:** Writing – review & editing, Visualization, Validation, Software, Project administration, Methodology, Formal analysis, Data curation, Conceptualization. **Fahao Shi:** Writing – review & editing, Visualization, Validation. **Wenjing Tang:** Writing – review & editing. **Zhou Wang:** Writing – review & editing. **Nazia Khalid:** Writing – review & editing, Software, Formal analysis. **Song Li:** Writing – review & editing. **Ana Chen:** Writing – review & editing, Visualization, Validation, Resources, Project administration, Methodology, Investigation, Funding acquisition.

## Funding

This work was supported by the Funding of Scientific Research Projects for Postdoctoral Researchers in Anhui Province (104/S012024013), Collaborative Innovation Project among Universities in Anhui Province (GXXT-2023-107), and the Key R&D project in Anhui Province (202204c06020004).

## Declaration of competing interest

The authors declare that they have no known competing financial interests or personal relationships that could have appeared to influence the work reported in this paper.

## Data Availability

Data will be made available on request.
